# SARS-COV-2 infections in inborn errors of immunity: A single center study

**DOI:** 10.3389/fimmu.2022.1035571

**Published:** 2022-11-21

**Authors:** Kimberley Cousins, Nicholas DeFelice, Stephanie Jeong, Jin Feng, Ashley Sang Eun Lee, Karina Rotella, David Sanchez, Faris Jaber, Shradha Agarwal, Hsi-en Ho, Charlotte Cunningham-Rundles

**Affiliations:** ^1^ The Division of Clinical Immunology, Department of Medicine, Icahn School of Medicine at Mount Sinai, New York, NY, United States; ^2^ The Division of Environmental Medicine and Public Health, Icahn School of Medicine at Mount Sinai, New York, NY, United States; ^3^ Institute of Exposomic Research, Icahn School of Medicine at Mount Sinai, New York, NY, United States; ^4^ Arnold Institute for Global Health, Icahn School of Medicine at Mount Sinai, New York, NY, United States

**Keywords:** coronavirus disease 2019 (COVID-19), severe acute respiratory syndrome coronavirus 2 (SARS-CoV-2), inborn errors of immunity (IEI), primary immunodeficiency (PID), common variable immunodeficiency (CVID), X-linked agammaglobulinemia (XLA), covid vaccination, monoclonal therapy

## Abstract

Severe acute respiratory syndrome coronavirus 2 (SARS-CoV-2) is a single-stranded RNA virus that causes coronavirus disease 2019 (COVID-19). One of the main topics of conversation in these past months in the world of immunology has been the issue of how patients with immune defects will fare if they contract this infection. To date there has been limited data on larger cohorts of patients with Inborn Errors of Immunity (IEI) diagnosed with COVID-19. Here, we review the data of COVID-19 infections in a single center cohort of 113 patients from the Mount Sinai Immunodeficiency program, who had 132 infections between January 2020 and June 2022. This included 56 males and 57 females, age range 2 - 84 (median 42). The mortality rate was 3%. Comparison between admitted patients revealed a significantly increased risk of hospitalization amongst the unvaccinated patients, 4% vaccinated vs 40% unvaccinated; odds ratio 15.0 (95% CI 4.2 – 53.4; p <0.00001). Additionally, COVID anti-spike antibody levels, determined in 36 of these patients post vaccination and before infection, were highly variable.

## Introduction

In December 2019, a novel single-stranded RNA Coronavirus, SARS-CoV-2, emerged in Wuhan, China (1). SARS-CoV-2 enters human cells *via* the angiotensin-converting enzyme-2 receptor expressed predominantly in the lung and intestinal epithelial cells, alveolar cells, and vascular endothelial cells and is mainly transmitted by exposure to infectious respiratory fluids (2, 3). SARS-CoV-2 causes an infectious disease with variable presentation ranging from a mild common cold to severe respiratory failure (3, 4). In Spring of 2020, New York City became the epicenter of the severe acute respiratory syndrome coronavirus 2 (SARS-CoV-2) pandemic (5). As of July 2022, a total of 564.1 million confirmed cases globally and 6,371,354 cumulative deaths had been reported by the World Health Organization (WHO) (6). Since emerging, COVID-19 has remained an ongoing global pandemic (5). An increased risk for severe disease and death has been noted among elderly patients and persons with preexisting medical conditions (7). Human inborn errors of immunity (IEI) otherwise referred to as primary immunodeficiency (PID) result from monogenic germline mutations, resulting in loss or gain of function of the encoded protein (8). In patients with IEI, the course of COVID-19 may vary from asymptomatic, to death; however, overall, there has been demonstration of high morbidity and mortality relative to the general population (9–11). In one study, 63% of 94 patients with primary immune deficiency who developed COVID-19, required hospitalization, with case-fatality rate of approximately 10% (12). In addition to limited studies on COVID-19 in IEI population, vaccine efficacy remains unclear. Data regarding the effectiveness of anti–SARS-CoV-2 vaccine in 26 patients with IEI, showed that the majority of patients were able to respond to vaccines, with levels of anti-SARS-CoV-2 IgG antibodies, ie. Anti-spike (anti-S) IgG antibodies, evaluated using commercial automated assay, as well as neutralizing antibodies, examined by angiotensin-converting enzyme 2 (ACE2)-RBD inhibition (ELISA), and cellular responses (13). However, 62 patients with predominantly antibody deficiency, characterized by low helper T cells, low B cells, and/or low class-switched memory B cells, were at risk for low antibody response to SARS-CoV-2 immunization, which however, improved following additional doses of vaccination in the majority of patients (10). The Mount Sinai Immune Deficiency program follows approximately 900 - 1,000 patients with primary immune deficiency, including infants referred for newborn screening for severe T cell defects, patients with agammaglobulinemia, a wide range of antibody defects, complement, T cell or neutrophil defects. In this study, we report the clinical course, follow-up, and outcome of COVID-19 in patients with IEI and correlate outcomes of disease with vaccination status.

## Materials and methods

### Patients and data collection

We analyzed a large cohort of IEI patients followed in the Mount Sinai Immune Deficiency program, who had a confirmed diagnosis of COVID-19, whether by PCR or rapid antigen testing, between March 2020 and July 2022. Spike-Antibody levels of a subset of these patients with IEI (n = 36) who had been vaccinated against SARS-CoV-2 before infection, were measured. Data including the patient demographics, clinical complications related to their IEI disease, treatments, antibody levels, vaccine status and outcomes were collected by either phone, chart review or at the time of in person follow up (**Table 1**). Data were collected and managed using Research Electronic Data Capture (REDCap) electronic data capture tools (14, 15). Statistical programing was done in MATLAB 2020a The MathWorks, Inc., Natick, Massachusetts, United States. This study was approved by the Institutional review board at Mount Sinai Medical Center.

**Table 1:** Clinical characteristics of patients with confirmed diagnosis of COVID-19 and IEI.

Parameter		n=113
Age (Median)		2-84 (42)
Sex	Male	56
	Female	57
Primary Immunodeficiency	CVID	63
	Agammaglobulinemia	15
	Hypogammaglobulinemia	15
	IgA Deficiency (with or without IgG2 Deficiency)	5
	Hyper IgM Syndrome	3
	Specific Antibody Deficiency	2
	Good Syndrome	2
	Kabuki Syndrome	1
	Di-George Syndrome	1
	APCED	1
	C5 Deficiency	1
	Hyper IgE Syndrome	1
	Wiskott-Aldrich Syndrome	1
	Activated PI3K Delta Syndrome	1
	Interferon gamma receptor deficiency	1
Co-morbidities	ITP	16
	Asthma	12
	Bronchiectasis	9
	Other (DM, thyroid disease, CAD, Obesity, kidney transplant)	9
	Solid Organ Cancer	6
	Hematologic Malignancy	8
	AIHA	5
	Nodular Regenerative Hyperplasia	2
	COPD	1

### Antibody determinations

Antibody testing (IgG anti SARS CoV-2 spike antibody), Elecsys^®^ Anti−SARS−CoV−2 for patients was done using the same commercial SARS-CoV-2 Semi-Quant Total Ab Chemiluminescent Immunoassay (CLIA). Results reported until May 2022 were between 0.8-2500 U/mL.

## Results

### Patient characteristics

**Figure 1:**
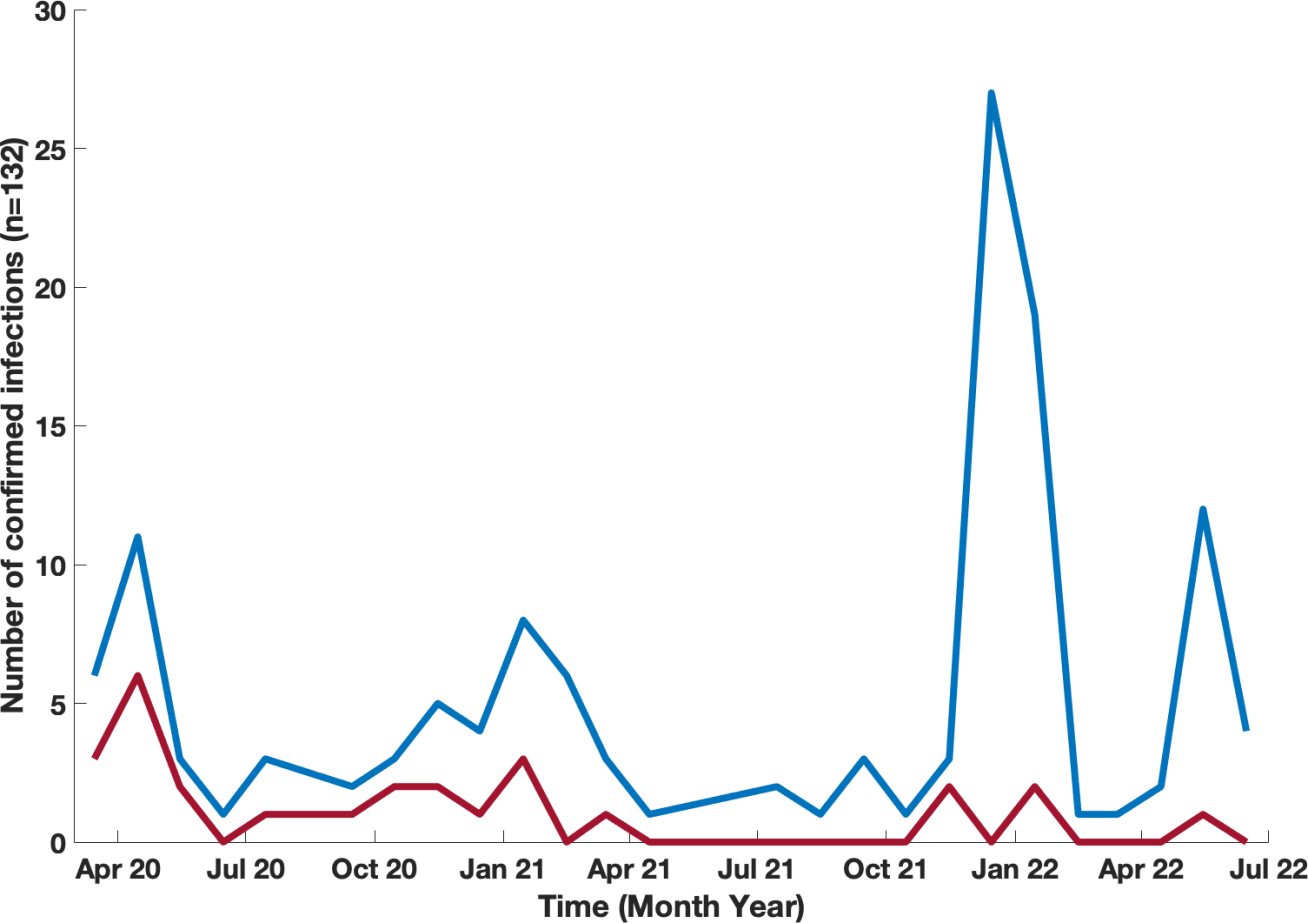
Timeline of confirmed COVID-19 infections in our patients with IEI. Month-by-month data of confirmed COVID-19 infections presented for patients who were infected blue line, and infected and hospitalized red line.

**Figure 2:**
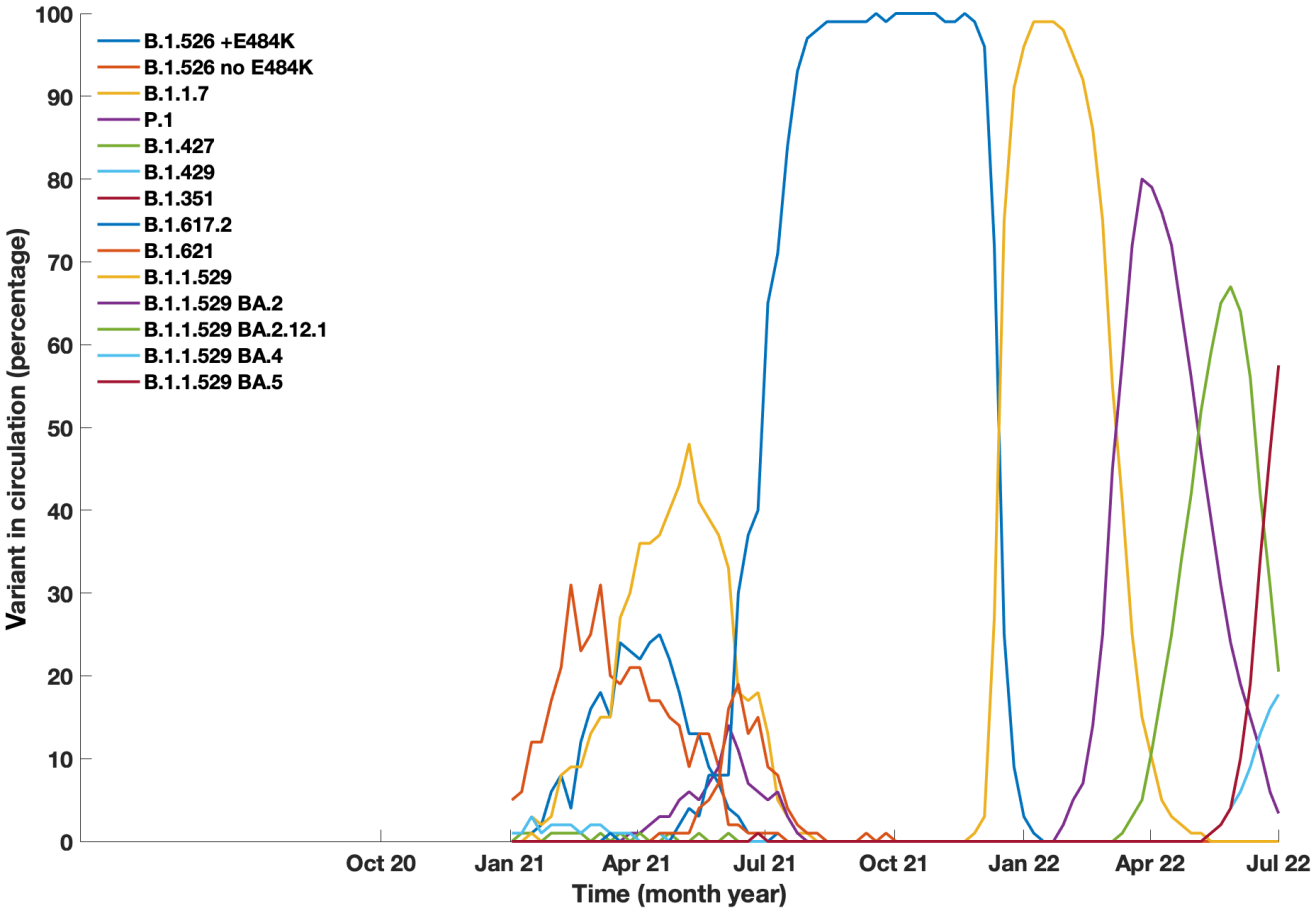
Prevalance of COVID-19 variants in circulation in New York City from January 2021 to July 2022. Data generated from Coronavirus Disease 2019 (COVID-19) in New York City (NYC), from the NYC Department of Health and Mental Hygiene https://github.com/nychealth/coronavirus-data.

**Figure 3:**
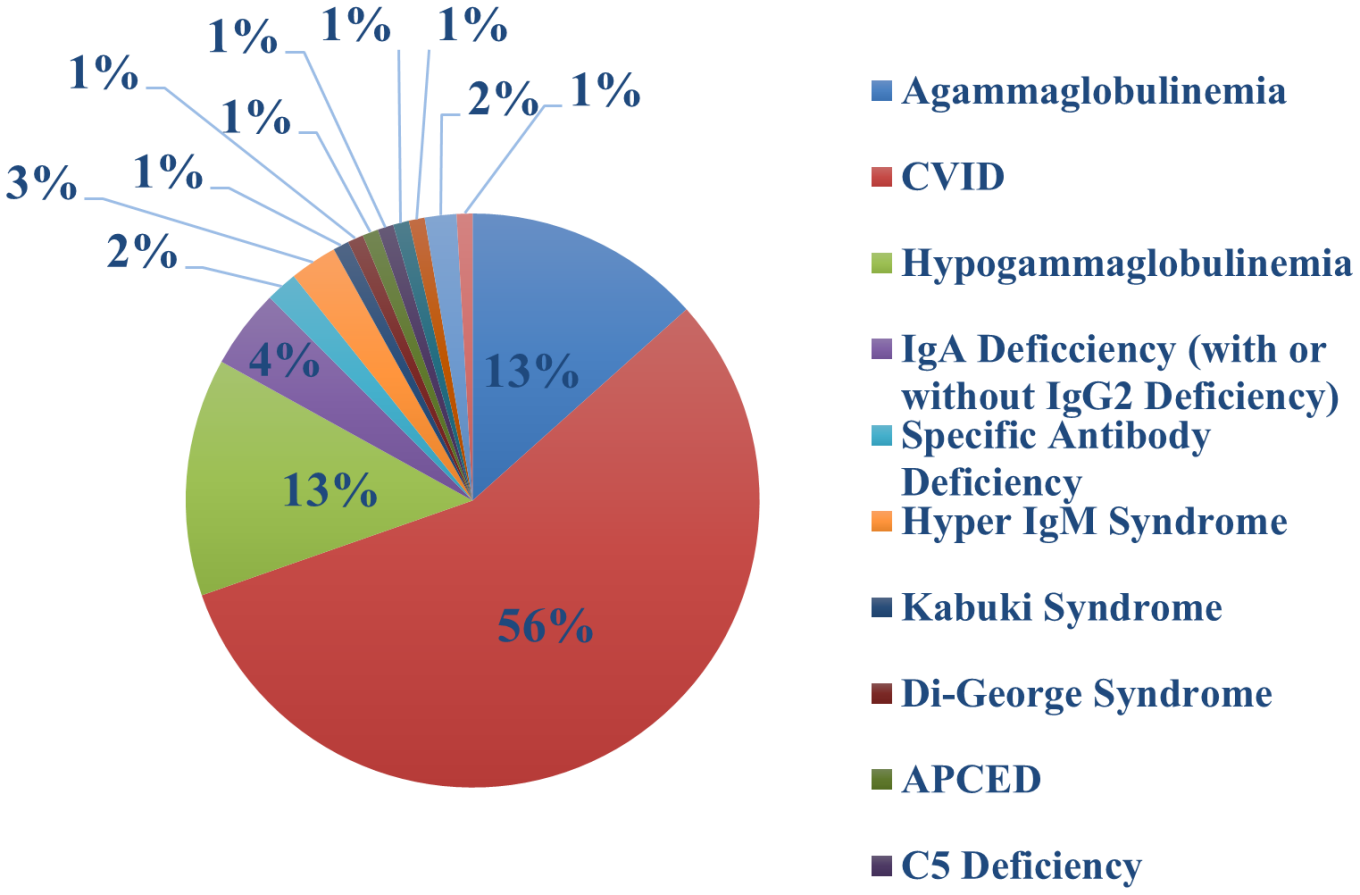
Diagnosis of immune defects in patients with COVID-19 infection.

One hundred and thirteen of our patients had a total of 132 confirmed Sars-COV-2 infections. The first recorded infection was in March 2020. The peak number of cases was observed in December 2021 when 27 new infections occurred (**Figure 1**); this coincided with the Omicron variant wave (BA.1.1529) in New York City (16). (**Figure 2**). The infected patients included 56 males and 57 females, of age range 2 to 84, (median age 42). The clinical parameters and course of COVID-19 in this cohort are shown (**Tables 1**, **2**). Fifteen patients had agammaglobulinemia (13 had X-Linked agammaglobulinemia, XLA), 63 patients had common variable immunodeficiency (CVID), 15 had hypogammaglobulinemia, 5 had IgA deficiency (with or without IgG2 deficiency), 2 had specific antibody deficiency, and 3 had hyper IgM syndrome. The other patients had Kabuki syndrome, Di-George Syndrome, autoimmune polyendocrinopathy-candidiasis-ectodermal dystrophy (APCED), complement C5 deficiency, hyper IgE syndrome, Wiskott-Aldrich, Good syndrome, activated PI3K Delta Syndrome and a homozygous IFN gamma R1 mutation (**Table 1**, **Figure 3**). Comorbidities summarized in (**Table 1**) were as follows; autoimmune hemolytic anemia (AIHA) (n = 5), asthma (n = 12), bronchiectasis (n = 9), chronic obstructive pulmonary disease (COPD) (n = 1), immune thrombocytopenic purpura (ITP) (n = 16), hemolytic malignancy (n = 8), solid organ malignancy (n = 6), and nodular regenerative hyperplasia (n = 2). Nine of these patients had other comorbidities, including diabetes mellitus, thyroid disease, coronary artery disease, obesity and organ transplant.

**Table 2:** Summary of management and outcomes of patients diagnosed with COVID-19 and IEI.

Parameter		Total number of infections (n = 132)
Vaccine status	Vaccinated	73
	Unvaccinated	59
Highest Level of Care	Outpatient	104
	Ward	23
	ICU	5
Respiratory Support	Supplemental Oxygen	17
	Intubation/ventilatory support	6
Treatment(s)	Monoclonal Antibodies	30
	Convalescent Plasma	19
	Azithromycin, HCQ	14
	Paxlovid	14
	Steroid (Dexamethasone)	11
	Remdesivir	11
	Tocilizumab	0
Prophylactic monoclonal (Evusheld)	–	1
Outcome	Died	4
	Recovered	128

### Infections and vaccinations

Of the 132 confirmed infections, 17 patients had two separate infections and one patient (with XLA) was infected on 3 separate occasions. The most commonly recorded symptoms with this illness were fever (84 of 132), dry cough (76 of 132), fatigue/weakness (38 of 132), dyspnea (35 of 132), congestion (31 of 132), diarrhea (7 of 16), rhinorrhea (20 of 132), headache (20 of 132), chills (18 of 132), diarrhea (18 of 132) and loss of smell/taste (12 of 132).

**Figure 4:**
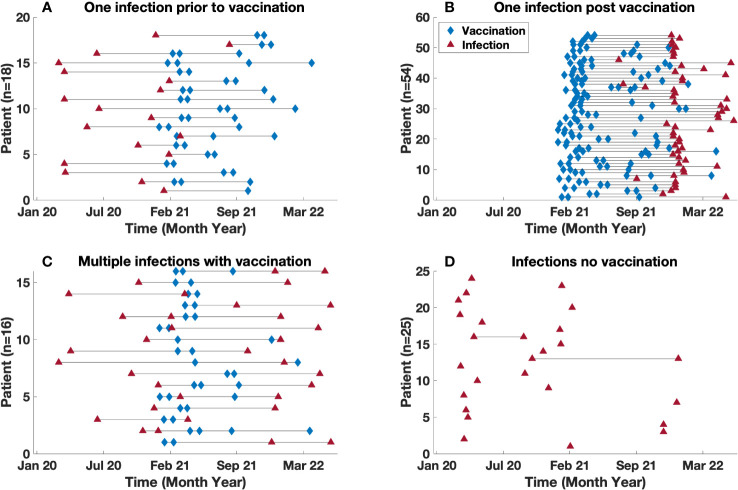
Time between vaccination and infection. Timeline demarcated by: **(A)** Single infection diagnosed prior to vaccination **(B)** Single infection diagnosed post vaccination **(C)** Multiple infections in vaccinated patients. **(D)** Infection(s) in unvaccinated population. Note, **(C)** includes patients with multiple infections both pre and post infection. The blue diamond and red triangle represents a vaccination and infection time point respectively.

Vaccinations for COVID-19 became available, starting with BNT162b2 (Pfizer) (17) which was granted emergency use authorization (EUA) on December 11, 2021. Shortly after this, the mRNA-1273 (Moderna) (18), and Ad26.COV2.S (Johnson and Johnson) (19) vaccines were granted EUA. In our cohort, 88 patients were vaccinated, while 25 patients declined. Of the 88 vaccinated subjects, 68 patients were infected at least once after vaccination leading to a total of 73 infections. Examining the timing of infections and vaccinations for these subjects, 18 of the vaccinated subjects had one infection prior to being vaccinated (**Figure 4A**), while 54 other patients had only one episode of COVID-19 post vaccination, with a mean time to infection post vaccination of 185 days (range 22 – 470) (**Figure 4B**). In contrast, 16 other vaccinated patients had infections both before and after vaccination. For this group of patients, the mean time for reinfection from most recent vaccination was 178 (26 – 294) days, and the mean time from infection to reinfection was 227 (range 46 – 440) days **Figure 4C**. For the 25 subjects who had declined vaccination, there were 27 infections, (two with re-infections) with a mean time between infections of 294 days (range 149 – 439) (**Figure 4D**).

### Treatments and outcomes

Of the 132 infections 104 cases were managed on an outpatient basis, while 28 patients were admitted to the hospital ward; 16 of these required supplemental oxygen therapy, five patients required the highest level of care in the intensive care unit and were intubated (**Table 2**). Infected patients received azithromycin, and/or hydroxychloroquine (n = 14), early in the pandemic. Other treatments included convalescent plasma (n = 19), remdesivir (n = 11) dexamethasone (n = 11). When this became available, 28 people received monoclonal antibody therapy for COVID-19 (infusions included Bamlanivimab/Etesevimab, Casirivimab/Imdevimab and Sotrovimab). Of the patients who received monoclonal therapy, 3 patients were hospitalized and 25 continued management as outpatients post infusion. After January 14, 2021, 62 additional infections were confirmed, however, did not receive monoclonal therapy upon diagnosis. Four out of 62 infections were hospitalized (**Table 2**). There was no statistical difference regarding outcomes for hospitalization with monoclonal therapy; odds ratio 0.6 (95% CI 0.1 – 2.8). When approved, Paxlovid was administered to 14 patients.

**Figure 5:**
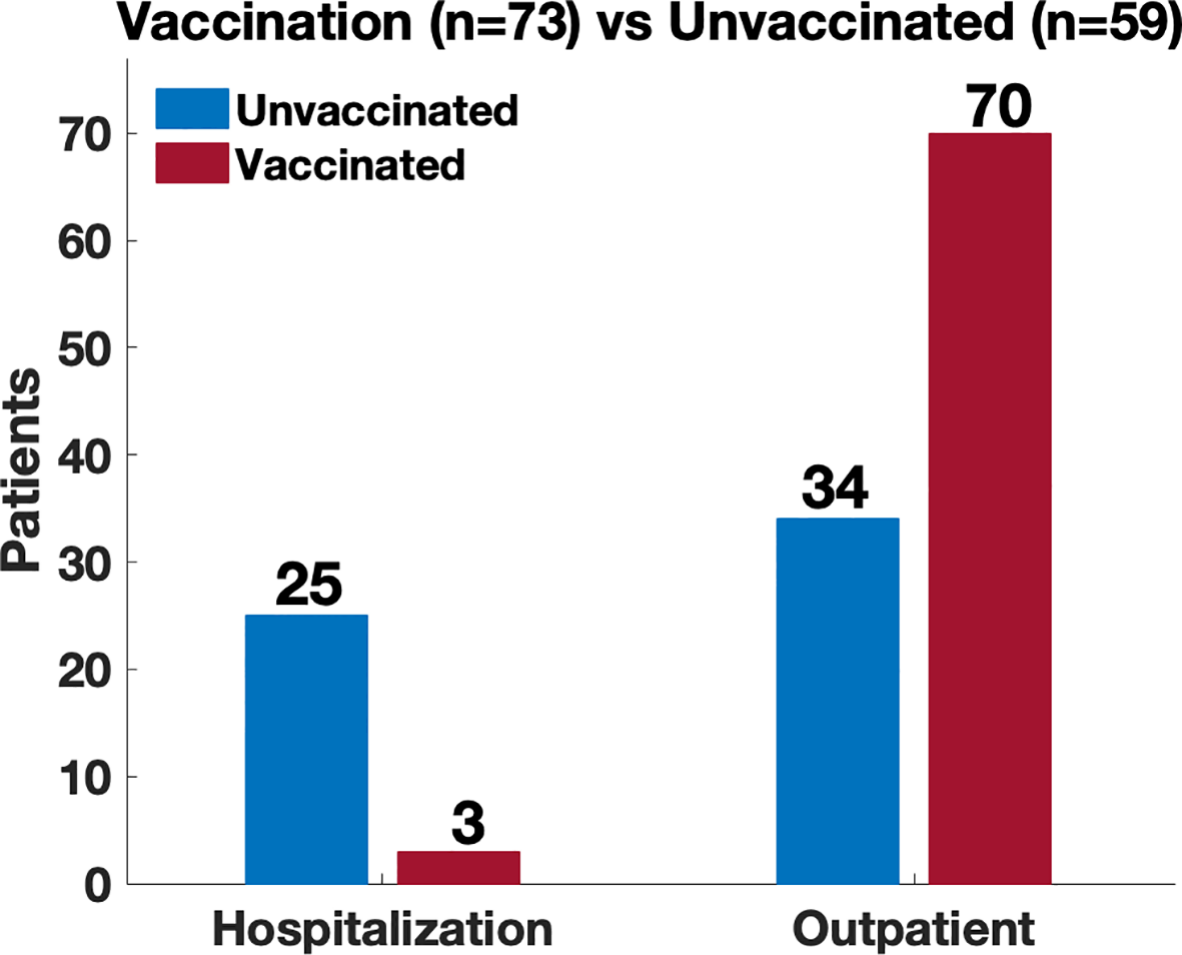
Comparison of disease severity between vaccinated and unvaccinated patients. Location of management (hospitalization vs outpatient) used as a proxy of disease severity. Comparison revealed a significantly increased risk of hospitalization amongst the unvaccinated patients (4% vaccinated vs 40% unvaccinated; odds ratio 15.0 (95% CI 4.2 – 53.4; p <0.00001)).

Sixty-eight patients had been vaccinated for COVID-19 prior to infection with SARS-CoV-2. Sixty-three of these received 2 doses as a part of the initial series for Moderna and Pfizer mRNA vaccines, while 5 patients received one dose of the Johnson and Johnson vaccine). Within the cohort of 68 patients who were infected post vaccination, there were 73 infections; of these, 3 patients required hospital admission, and all required supplemental oxygen therapy. A comparison between admitted patients revealed a significantly increased risk of hospitalization in the unvaccinated patients [4% vaccinated vs 40% unvaccinated; odds ratio (OR) 15.0 (95% CI 4.2 – 53.4; p <0.00001)] (**Figure 5**). There were no ICU admissions in the vaccinated cohort. Also, during the time period after vaccinations became available (January 2021), there were also 20 patients who recorded an infection and remained unvaccinated, 6 of these patients were admitted to the hospital (3 of whom were age <15).

We analyzed the need for hospitalization by underlying diagnosis of IEI, separating patients with predominant antibody deficiencies [including agammaglobulinemia, CVID, hypogammaglobulinemia, IgA deficiency (with or without IgG2 deficiency), specific antibody deficiency, hyper IgM syndrome or Good syndrome] from those the other diagnosis encountered [Kabuki syndrome, Di-George syndrome, APCED, C5 deficiency, hyper IgE syndrome, Wiskott-Aldridge syndrome, activated PI3K delta syndrome or interferon gamma receptor deficiency] (**Table 3**). We noted that patients with these primarily antibody deficiencies were 14 times more likely to be hospitalized if infected while unvaccinated as compared to after vaccination. (OR 14.7 (95% CI 4.1 – 52.8); p <0.000001). Those with the other diagnoses were 4.5 more likely to be hospitalized if infected while unvaccinated as compared to those who had been vaccinated OR 4.5 (95% CI 0.2 – 80.6; p < 0.5, however this was not statistically significant).

**Table 3:** Summary of outcomes of patients diagnosed with COVID-19 based on IEI diagnosis.

	Unvaccinated				Vaccinated	
Primary immunodeficiency	Outpatient	Hospitalization	ICU	Death	Outpatient	Hospitalization
Predominant antibody deficiency						
Agammaglobulinemia	4	8	–	–	7	1
CVID	21	5	1	3	40	1
Hypogammaglobulinemia	4	–	–	–	12	–
IgA deficiency (with or without IgG2 Deficiency)	1	–	–	1	4	1
Specific antibody deficiency	1	–	–	–	1	–
Hyper IgM syndrome	1	2	–	–	2	–
Good syndrome	–	1	–	–	1	–
Other diagnoses						
Kabuki syndrome	–	1	–	–	–	1
Di-George syndrome	1	–	–	–	–	–
APCED	–	–	–	–	1	–
C5 seficiency	–	–	–	–	1	–
Hyper IgE syndrome	–	1	–	–	–	–
Wiskott-Aldrich syndrome	1	–	–	–	–	–
Activated PI3K delta syndrome	–	–	–	–	1	–
Interferon gamma receptor deficiency	–	1	–	–	–	–

The majority of infected patients survived, and fully recovered from acute disease. Four patients, early in the pandemic died from COVID-19 (CVID, n = 2; hypogammaglobulinemia, n = 1; IgA-IgG2 deficiency, n = 1) (5). Three of the patients who died had preexisting immune deficiency-associated autoimmune/inflammatory complications; 2 of these also had preexisting IEI-associated chronic lung disease (bronchiectasis, n = 1; interstitial lung disease, n = 1). In addition, one patient was a kidney transplant recipient who had previously had lymphoma. The age of the patients who died ranged from 39 to 76 years (5) (20). None had been vaccinated, as this was before the FDA authorized vaccines became available in January 2021. The five patients who were intubated and required intensive care unit level care were also unvaccinated.

### Antibody levels

**Figure 6:**
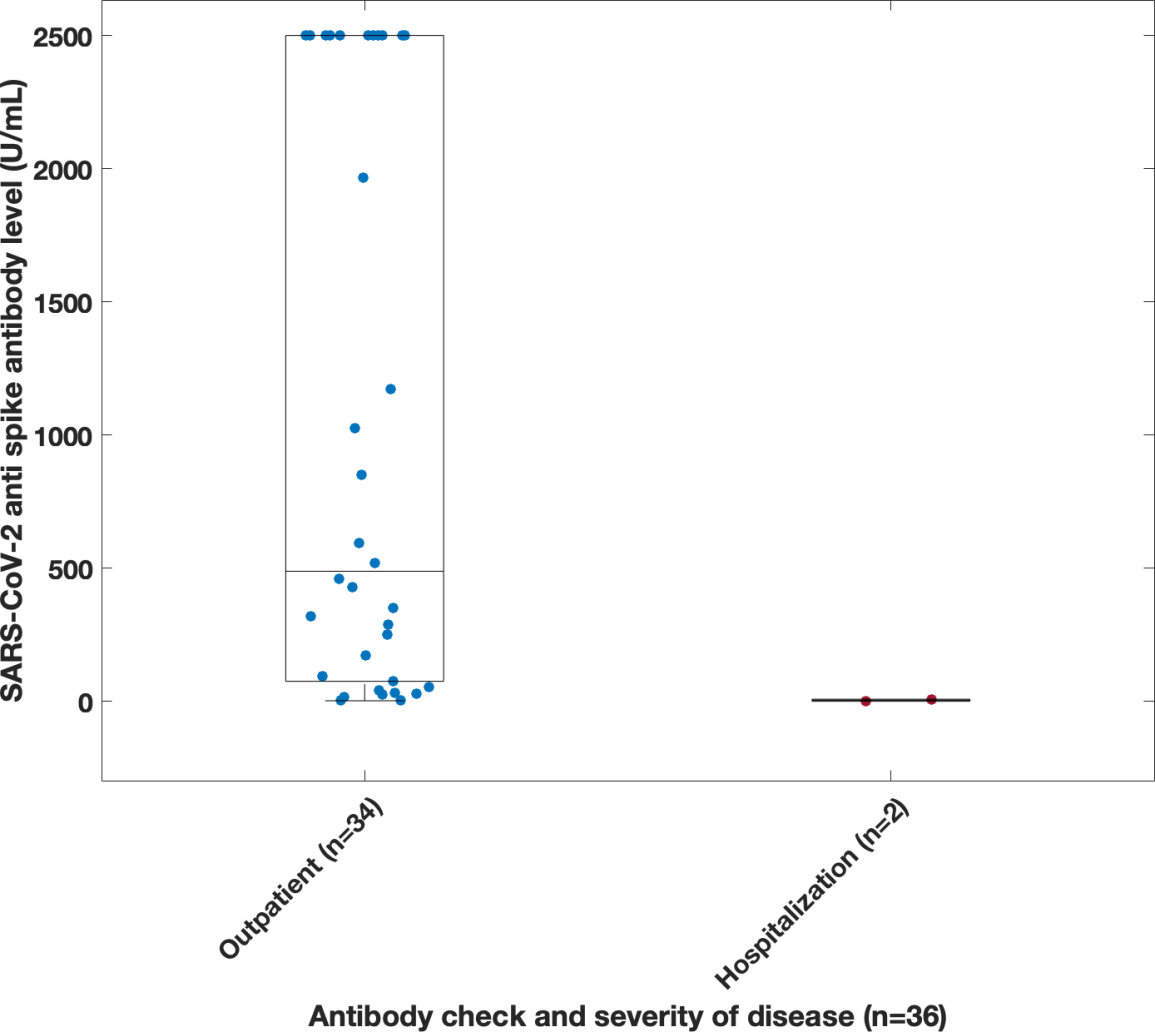
Correlation between disease severity and spike antibody level taken prior to infection. Antibodies were derived from multiple source including vaccination, immunoglobulin product, monoclonal infusions and convalescent plasma. Note, spike antibody test limit was 2500 U/ml.

Thirty-six of the 58 patients **(** **Figures 4B, C** **)** who were not infected prior to vaccination, had serum COVID anti-spike antibody levels checked prior to their confirmed infection (**Figure 6**). The amounts varied widely from 0 to 2500 U/mL. The antibody present in serum of these patients represents vaccination responses and/or contributions from immunoglobulin treatments, as this antibody began to appear in Ig products this interval (21, 22). There was no correlation between antibody level and need for hospitalization, based on the available data. However, it was observed that 2 of the hospitalized patients had an antibody level of < 10 U/ml. Both patients (ages 46 and 69) had a diagnosis of agammaglobulinemia (one with confirmed XLA), and one had known lung disease (bronchiectasis).

## Discussion

The Mount Sinai Immunodeficiency Program previously reported the first group of 16 patients who developed COVID-19 early in the pandemic. The proportion of deaths in the original series (25%) was greater than that in the general population with COVID-19 reported at New York City hospitals (10.2%) at that time (5). From July 2020 to November 2021, the incidence of additional cases in our cohort remained relatively low, with 21% of cases occurring in that time range (**Figure 1**). This was perhaps due to behavioral practices such as masking, social distancing, and strict self-isolation. However, as variants such as the Omicron variant (BA.1) began to circulate (16), the infection rate began to rise in late 2021. Although the number of reported infections increased, we did not observe a large increase in rates of hospitalization with subsequent variants. In fact, there were no additional mortalities after the initial 4 patients reported (5). This was likely due to differences in viral strains, large variation in age range within our cohort, improved treatment measures, availability of convalescent plasma, monoclonal antibody infusions, and prophylactic therapies such as monoclonal infusions and vaccinations. Although analyses of monoclonal therapy in these patients revealed no statistical differences regarding outcomes for hospitalizations (odds ratio 0.6 (95% CI 0.1 – 2.8)) this could be due to a smaller size cohort of patients receiving this therapy, and/or patients having additional protection from vaccination. Additionally, the monoclonal antibodies available earlier in the pandemic no longer demonstrated the same efficacy with regard to later circulating variants (23).

For this cohort, there was an overall mortality rate of 3%; However, all deaths were early in the pandemic (5). Even though 3% is lower compared to other cohorts reported, with inpatient mortality rate at 40% (24), the 3% mortality rate seen in our cohort would still represent a high morbidity and mortality relative to the general population (1.1% in the US) (9–12, 25). Other studies had similar findings seen in our cohort, where patients through different phases of the pandemic experienced a mild clinical course with limited symptoms (26). Higher mortality in earlier studies could be explained by differences in viral strains, and additional modalities of treatment including use of steroids, plasma, antivirals, and monoclonal infusions for confirmed infections as well as prophylaxis with vaccines and monoclonal infusions.

Thirty-six patients had anti spike antibody levels evaluated prior to infection; these levels were highly variable and revealed no correlation with prevention of hospitalization, demonstrating that the level, and/or value of the antibodies tested is far from clear. Neutralizing antibodies were not examined. In one study, immune deficient patients showed a wide range of anti-spike antibody titers, ranging from undetectable levels to normal-to-high titers. In fact, when patients with XLA were excluded, 18 of 22 of the examined patients (81.8%) tested positive for anti-S antibodies (13). However, in another study, subjects with antibody deficiency were found to have lower mean anti-spike antibody levels, compared to matched healthy controls, leading to the assumption that patients were less protected (10). In addition to antibody, the role of T cell responses may also be critical components of the immune protection against SARS-CoV-2 (27–29).

Comparisons between admitted patients revealed a significantly increased risk of hospitalization amongst the unvaccinated patients [4% vaccinated vs 40% unvaccinated; odds ratio 15.0 (95% CI 4.2 – 53.4; p <0.00001)] (**Figure 5**) which suggest that vaccination may provide some level of protection against severe disease (10). This may be due to the generation of neutralizing antibody as well as augmented T cell responses. In one study, vaccines induced a strong cellular response with a trend toward statistical significance when convalescent individuals with IEI and no prior history of infection were compared with healthy vaccinated donors (13).

In summary, the clinical impact of COVID-19 in patients with primary immune deficiency varies from mild symptoms to death. The proportion of deaths in this series (3%) was greater than that in the general population with COVID-19 reported in New York (0.28% crude mortality rate) and in the US (1.1% mortality rate) (25, 30). However, over time, the patients who have become infected, have been less ill and have required few hospitalizations, similar to that of the general population, potentially due to less virulent variants of SARS-CoV-2 (31). However, the role of additional treatments such as our early use of plasma (32), and more recently, monoclonal antibodies and Paxlovid have been essential therapies.

## Data availability statement

The raw data supporting the conclusions of this article will be made available by the authors, without undue reservation.

## Ethics statement

The studies involving human participants were reviewed and approved by Institutional review board at Mount Sinai Medical Center. Written informed consent to participate in this study was provided by the participants’ legal guardian/next of kin.

## Author contributions

All authors listed in this research contributed to the final publication. KC main author and primary investigator designed and executed the project, NF designed algorithms in MATLAB to process data and participated in compiling the paper, SJ participated in data collection, JF participated in data collection, AL participated in data collection, KR participated in data collection, DS participated in data collection, FJ participated in data collection, SA contributed to writing and review of the paper, H-eH contributed to writing and review of the paper, CC-R is the Senior investigator of the project.

## Conflict of interest

The authors declare that the research was conducted in the absence of any commercial or financial relationships that could be construed as a potential conflict of interest.

## Publisher’s note

All claims expressed in this article are solely those of the authors and do not necessarily represent those of their affiliated organizations, or those of the publisher, the editors and the reviewers. Any product that may be evaluated in this article, or claim that may be made by its manufacturer, is not guaranteed or endorsed by the publisher.
